# Fabrication and Characterization of Strontium-Substituted Hydroxyapatite-CaO-CaCO_3_ Nanofibers with a Mesoporous Structure as Drug Delivery Carriers

**DOI:** 10.3390/pharmaceutics10040179

**Published:** 2018-10-08

**Authors:** Shiao-Wen Tsai, Wen-Xin Yu, Pai-An Hwang, Sheng-Siang Huang, Hsiu-Mei Lin, Yu-Wei Hsu, Fu-Yin Hsu

**Affiliations:** 1 Graduate Institute of Biomedical Engineering, Chang Gung University, Taoyuan City 33302, Taiwan; swtsai@gap.cgu.edu.tw; 2 Department of Orthopedic Surgery, Chang Gung Memorial Hospital, Linko 33305, Taiwan; 3 Department of Periodontics, Chang Gung Memorial Hospital, Taipei 10507, Taiwan; 4 Department of Bioscience and Biotechnology, National Taiwan Ocean University, Keelung City 20224, Taiwan; andy54861@yahoo.com.tw (W.-X.Y.); amperehwang@ntou.edu.tw (P.-A.H.); mm0070360@gmail.com (S.-S.H.); hmlin@mail.ntou.edu.tw (H.-M.L.); qazwest74@gmail.com (Y.-W.H.)

**Keywords:** strontium, hydroxyapatite, mesoporous, drug delivery

## Abstract

Hydroxyapatite (HAp) is the main inorganic component and an essential part of hard bone and teeth. Due to its excellent biocompatibility, bioactivity, and osteoconductivity, synthetic HAp has been widely used as a bone substitute, cell carrier, and therapeutic gene or drug carrier. Recently, numerous studies have demonstrated that strontium-substituted hydroxyapatite (SrHAp) not only enhances osteogenesis but also inhibits adipogenesis in mesenchymal stem cells. Mesoporous SrHAp has been successfully synthesized via a traditional template-based process and has been found to possess better drug loading and release efficiencies than SrHAp. In this study, strontium-substituted hydroxyapatite-CaO-CaCO_3_ nanofibers with a mesoporous structure (mSrHANFs) were fabricated using a sol–gel method followed by electrospinning. X-ray diffraction analysis revealed that the contents of CaO and CaCO_3_ in the mSrHANFs decreased as the doping amount of Sr increased. Scanning electron microscopy (SEM) images showed that the average diameter of the mSrHANFs was approximately 200~300 nm. The N_2_ adsorption–desorption isotherms demonstrated that the mSrHANFs possessed a mesoporous structure and that the average pore size was approximately 20~25 nm. Moreover, the mSrHANFs had excellent drug- loading efficiency and could retard the burst release of tetracycline (TC) to maintain antibacterial activity for over 3 weeks. Hence, mSrHANFs have the potential to be used as drug carriers in bone tissue engineering.

## 1. Introduction

The development of multifunctional bioactive materials for the repair of bone defects caused by tumor resection, infections, trauma, or skeletal abnormalities is an important issue [1,2,3]. Bone defects caused by infection are difficult to regenerate due to the difficulty in eradicating pathogens. Surgical debridement and long-term systemic antibiotic therapies are the main treatment methods for chronic bone infection. However, long-term administration of antibiotics can cause antibiotic resistance. Hence, bone grafts possessing the capacity to simultaneously release antibiotics in a localized and sustained manner and stimulate bone regeneration for repairing bone defects caused by osteomyelitis are desirable [4].

Hydroxyapatite (HAp), the main mineral component of bone and tooth, is widely used as a bone graft due to its excellent biocompatibility, bioactivity, and osteoconductivity. The sol–gel method offers molecular-level mixing of calcium and phosphorus precursors under very mild conditions for synthesizing HAp nanoparticles and can ameliorate the chemical homogeneity of HAp. Doping HAp with various ions, such as magnesium, manganese, zinc, and strontium, can alter important physicochemical properties such as surface reactivity, thermal stability, and solubility rate [5,6,7,8]. Zhang et al. [9] pointed out that hydroxyapatites obtained by partial substitution of Ca^2+^ by Sr^2+^ have a higher solubility than pure HAp due to the ionic radius difference (Ca^2+^ = 0.100 nm, Sr^2+^ = 0.118 nm), which results in a general perturbation of the crystal lattice. Strontium ranelate, a strontium (II) salt of ranelic acid, has been demonstrated to have the ability not only to promote the osteogenic differentiation of mesenchymal stem cells but also to inhibit the differentiation of osteoclasts [10,11]. Sr ions play an important role in both the stimulation of bone formation and the reduction in bone resorption during bone remodeling [12]. Previous in vitro and in vivo studies have demonstrated the osteogenic effect of strontium-substituted hydroxyapatite (SrHAp) [13,14].

Mesoporous materials have attracted much attention as drug delivery systems because of their excellent drug loading capacity and drug release efficacy [15,16]. Recently, mesoporous SrHAp nanoparticles have been synthesized via the sol–gel process by using cetyltrimethylammonium bromide (CTAB) as a template reagent [17,18]. Zhang et al. [19] synthesized SrHAp nanorods with mesoporous properties and found that ibuprofen molecules can be adsorbed onto the surface of the mesoporous SrHAp nanorods in the impregnation process and released by a diffusion-controlled mechanism.

Ramanan et al. [20] fabricated hydroxyapatite fibers by spinning sols prepared from a 2-butanol solution of phosphorous pentoxide and calcium acetate in aqueous solution in a rotating spinneret system. Franco et al. [21] produced HAp nanofibers by combining electrospinning with a sol–gel system using phosphorous pentoxide and calcium nitrate tetrahydrate as precursors in ethanol solution. Electrospinning is a versatile bottom-up technique for fabricating porous three-dimensional scaffolds with nanofibrous structures mimicking the native extracellular matrix (ECM) [22].

In our previous work, we fabricated mesoporous hydroxyapatite-CaO composite nanofibers (p-HApFs) via the sol–gel process by using Pluronic P123 as a porogen and an electrospinning technique and found that the p-HApFs possessed good drug loading efficiency and could retard the burst release of tetracycline (TC). [23] The formation of CaO may have been due to the incomplete pyrolysis of Pluronic P123 and the absorption of CO_2_ from the atmosphere during the calcination process. Lopatin et al. have also reported a similar result regarding the formation of CaO in the sol–gel processing of HAp [24]. However, many studies have noted that CaO has a negative effect on the biocompatibility of HAp [25,26]. Kanchana demonstrated that the addition of strontium to the synthesis of biphasic calcium phosphate using the sol–gel method could decrease the formation of CaO impurities [27].

Sr-HAp has been commonly used in either bulk or powder form. Several studies have noted that cells interact more strongly with nanofibers [28,29]. This study aimed to fabricate and characterize strontium-substituted hydroxyapatite-CaO-CO_3_ nanofibers with a mesoporous structure (mSrHANFs) through an electrospinning process based on a sol–gel precursor with CTAB as the porogen and to evaluate the effect of different amounts of Sr doping on the components of mSrHANFs. In addition, the drug loading efficiency, mechanism of drug release, and antibacterial activity of tetracycline (TC) released from the mSrHANFs were assessed.

## 2. Materials and Methods

### 2.1. Reagents

Calcium nitrate tetrahydrate, strontium nitrate, cetyltrimethylammonium bromide (CTAB), poly(ethylene glycol)-poly(propylene glycol)-poly(ethylene glycol) (Pluronic P123, MW = 5800) and poly(vinyl pyrrolidone) (PVP, MW = 40,000), and tetracycline hydrochloride (TC) were purchased from Sigma-Aldrich Chemical Company (St. Louis, MO, USA). Triethyl phosphite (TEP) was purchased from Merck (Darmstadt, Germany). All other chemicals used were reagent grade unless otherwise stated.

### 2.2. Synthesis and Characterization of Strontium-Substituted Hydroxyapatite-CaO-CO_3_ Nanofibers Containing with a Mesoporous Structure (mSrHANFs)

In this study, CTAB was used as a porogen to synthesize mSrHANFs. Briefly, 0.5 g of CTAB and 3.086 mL of TEP were mixed in 5 mL of ethanol aqueous solution (50% *v/v*) and continuously stirred until the solution was clear. Calcium nitrate was dissolved in 95% ethanol solution, and strontium nitrate was dissolved in deionized water at room temperature. Subsequently, the calcium nitrate and strontium nitrate solutions were slowly added dropwise to the above TEP/CTAB solution to form a precursor solution. The Sr/(Sr + Ca) molar ratios selected were 10, 20, and 30 mol%, whereas the (Sr + Ca)/P molar ratio was fixed at 1.67. Poly(vinyl pyrrolidone) (PVP) and Poly(ethylene glycol)-poly(propylene glycol)-poly(ethylene glycol) (Pluronic P123) were dissolved in 7 mL of absolute ethanol and incorporated into the 3 mL precursor solution after placing the precursor solution in an oven at 60 °C for 12 h. A nonwoven nanofiber film was fabricated by electrospinning. Basically, the precursor solution was added to a syringe fitted with a needle (18 G, inner diameter = 0.838 mm), and the technique was performed at a steady flow rate (1.27 mL/h) and an electrical field (1.3 kV/cm, Spellman SL 60^®^, New York, NY, USA). The polymer solution was ejected, and the formed nanofibers were collected on an aluminum substrate and then calcined at 800 °C under a nitrogen atmosphere to obtain the mSrHANFs. The sample notation and initial Sr/Ca molar ratios for the fabrication of the mSrHANFs are shown in Table 1.

### 2.3. Characterization of the mSrHANFs

The structure of the mSrHANFs was observed using scanning electron microscopy (SEM, ZEISS SIGMA, Dresden, Germany) and transmission electron microscopy (TEM, JEOL JEM-2100, Tokyo, Japan). The average diameter of the mSrHANFs was analyzed using SEM images with image analysis software (Image-Pro Express Version 6.0, Media Cybernetics, Rockville, MD, USA). The phase composition of the mSrHANFs was characterized by X-ray diffraction (XRD, Bruker D2-Phaser, Madison, WI, USA), SEM-energy dispersive spectrometry (SEM-EDS), and Fourier transform infrared spectroscopy (FTIR, Bruker tensor II, Madison, WI, USA). The nitrogen adsorption–desorption experiment was performed to obtain the Brunauer-Emmett-Teller (BET) specific surface area and pore size (Micromeritics ASAP 2020 instrument, Norcross, GA, USA). The chemical compositions of the mSrHANFs were analyzed by inductively coupled plasma–optical emission spectrometry (ICP-OES, Agilent Technologies, Santa Clara, CA, USA).

### 2.4. In Vitro Study of the Degradability of mSrHANFs

mSrHANFs (10 mg) were soaked in minimal essential medium (MEM, 1 mL) for 1 day. After soaking, the medium was centrifuged, and the supernatant solution was used to evaluate the released ions by ICP-OES. Because there was no strontium in MEM, the dissolution ratio (S) of the mSrHANFs was calculated by the following equation: S = [medium_Sr_/mSrHANFs_Sr_] × 100%, where medium_Sr_ and mSrHANFs_Sr_ were the Sr content in the MEM and mSrHANFs, respectively.

### 2.5. In Vitro Study of Drug Loading and Release

Tetracycline (TC) was selected as the model drug to measure drug loading efficiency and release profile. In brief, 3mSrHANFs (20 mg) was added to TC aqueous solution (10 mg/mL, 2 mL) and stirred for 24 h. The TC-loaded 3mSrHANFs were then collected by centrifugation, washed with deionized water, and freeze-dried. The amount of TC adsorbed onto the 3mSrHANFs was determined by the difference in TC concentration in the loading medium before and after the loading process. The concentration of TC was analyzed with a UV-Vis spectrophotometer (Ultrospec 1100 Pro, Amersham Biosciences, Piscataway, NJ, USA) at a wavelength of 360 nm.

The drug content and loading efficiency were calculated according to the following formula:

Drug content (*w*/*w*) = weight of TC in the 3mSrHANFs/weight of the 3mSrHANFs

Loading efficiency (%) = (weight of TC in the 3mSrHANFs/initial weight of TC) × 100%

TC-loaded 3mSrHANFs (20 mg) were lyophilized, then placed into phosphate-buffered saline (PBS) solution (2 mL) and agitated in a horizontal shaking bath at 37 °C. The release medium was withdrawn and replaced with fresh PBS at each measurement. 

### 2.6. Antibiotic Activity against Staphylococcus Aureus and Pseudomonas Aeruginosa

The antibiotic activity of TC in the in vitro release study was assessed against the Gram-positive bacteria *Staphylococcus aureus* and the Gram-negative bacteria *Pseudomonas aeruginosa*. A total of 100 μL of TC eluate from the in vitro release study or Lysogeny Broth growth medium (as a positive control) was added to an inoculum containing 100 μL of bacteria in a 96-well plate. After 24 h of incubation, the optical density (OD) of the culture medium was measured using a spectrophotometer at a wavelength of 600 nm. The relative antibacterial activity (R%) was calculated as follows:

$$\;R\%=\;\frac{ODgrowth\;medium-ODreleased\;tetracycline}{ODgrowth\;medium}\;\times \;100\%\;$$

## 3. Results and Discussion

**Figure 1:**
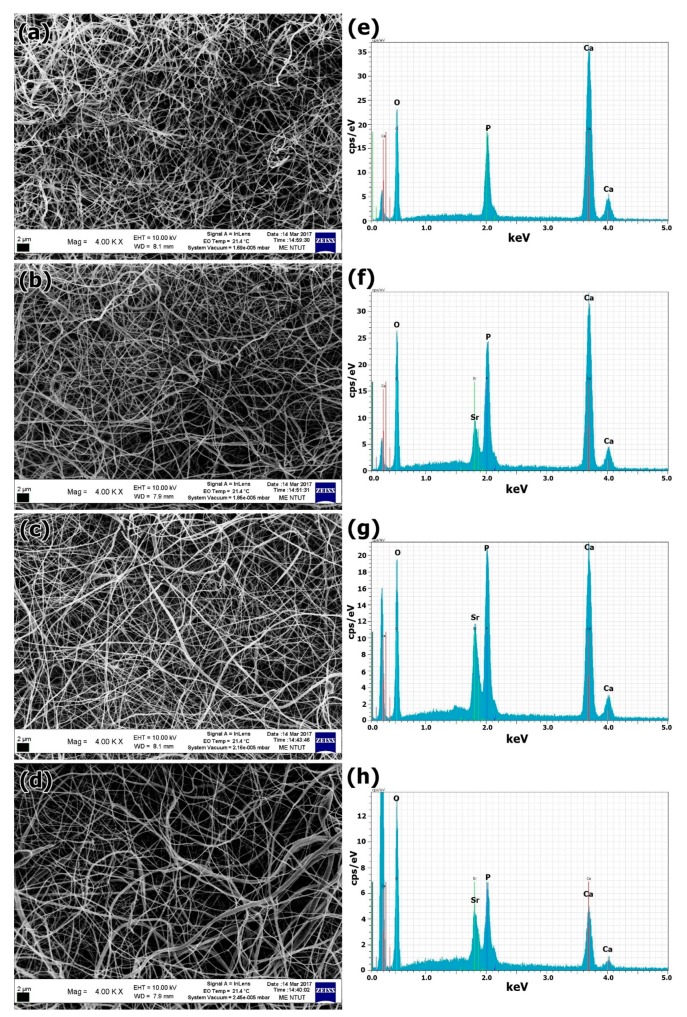
(**a**–**d**) Scanning electron microscope (SEM) micrographs and (**e**–**h**) Scanning electron microscopy-energy dispersive X-ray (SEM-EDX) spectra of the mSrHANFs. (**a**,**e**) 0mSrHANFs; (**b**,**f**) 1mSrHANFs; (**c**,**g**) 2mSrHANFs; (**d**,**h**) 3mSrHANFs.

The morphology and microstructure of the mSrHANFs were observed under SEM and TEM. Figure 1 shows SEM images of the mSrHANFs. Fiber diameters are reported as the median (1st quartile, 3rd quartile). The fiber diameters (nm) of the 0mSrHANFs, 1mSrHANFs, 2mSrHANFs and 3mSrHANFs were 200 (181, 226), 217 (175, 246), 231 (195, 264), and 240 (192, 289), respectively. The doping amount of Sr slightly increased the diameters of the nanofibers. 

The SEM-EDS analysis results of the mSrHANFs showed that the nanofibers were composed of only Ca, Sr, O, and P. Chemical analysis of the mSrHANFs shows a slight Sr^2+^ deficiency compared with the nominal values (shown in Table 2). Conversely, the (Sr + Ca)/P ratios of the 0mSrHANFs, 1mSrHANFs, 2mSrHANFs, and 3mSrHANFs from ICP-OES analysis were 3.17, 2.08, 1.73, and 1.68, respectively. The (Sr + Ca)/P ratio of the 3mSrHANFs is very close to the stoichiometric Ca/P ratio for pure HAp (1.67). 

**Figure 2:**
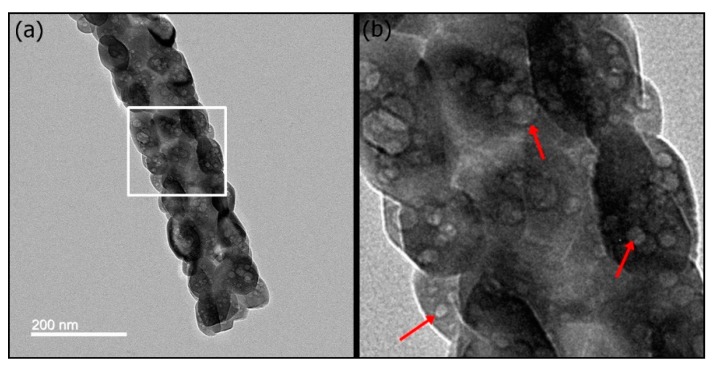
Transmission electron microscopy (TEM) micrographs of the 3mSrHANFs. The image in (**b**) showed an enlarged view of the white square in (**a**). The red arrows in (**a**) indicate the nonordered orientation of the mesopores within the nanocrystals.

Structure-directing agents such as CTAB, Pluronic F127, and Pluronic P123 play a key role in influencing the mesoporous structure and the pore size of mesoporous materials [30]. Pluronic P123-induced mesoporous bioactive glass (MBG) has an ordered, two-dimensional hexagonal mesoporous structure. In contrast, CTAB-induced MBG has less mesostructural ordering. In this study, CTAB-induced mSrHANFs showed a nonordered orientation of the mesopores within the nanocrystals (Figure 2). The microstructure is same as that observed in Pluronic P123-induced p-HApFs [23].

**Figure 3:**
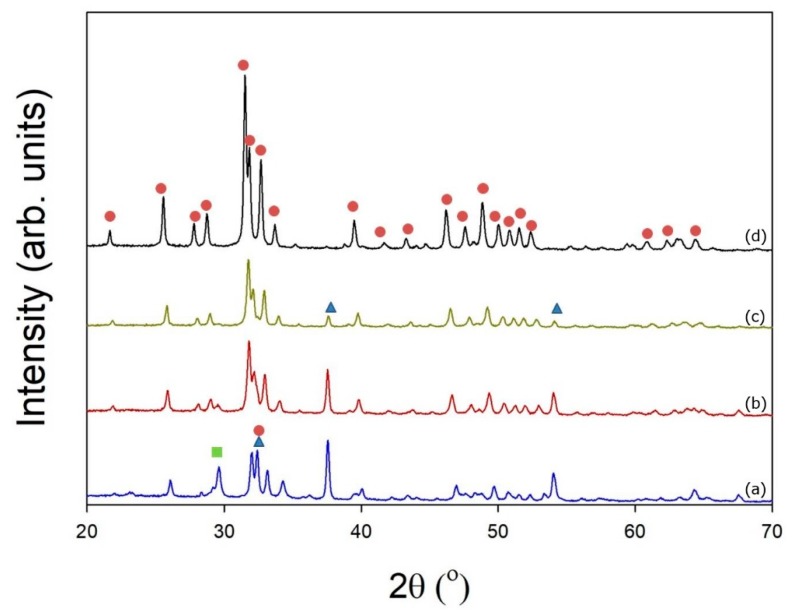
X-ray diffraction (XRD) patterns of (**a**) 0mSrHANFs, (**b**) 1mSrHANFs, (**c**) 2mSrHANFs, and (**d**) 3mSrHANFs. ( 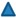: CaO; 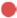: HAp/Sr-HAP; 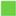: CaCO_3_).

The crystallographic structures of the mSrHANFs were determined by wide-angle XRD analysis. Diffractograms of all samples are reproduced in Figure 3. The characteristic diffraction peaks of HAp shift to lower 2θ values upon Sr addition, indicating an increase in the lattice constants [31] (Table 3). O’Donnell et al. [32] expected that because Sr is slightly larger (118 pm vs. 100 pm), heavier, and more electron rich than Ca, substitution of Ca with Sr would lead to increased d-spacings and more effective scattering of X-rays. Moreover, to determine the phase composition, spectra were analyzed with DIFFRAC.SUITE EVA software (Bruker v.4.2.) using Powder Diffraction File (PDF) cards #01-1160-calcium oxide, #85-1108-calcium carbonate, #760694-hydroxyapatite, #89-5631-Sr-substituted hydroxyapatite, and #89-5632-Sr-substituted hydroxyapatite as structural models. Table 4 reveals that the content of the CaO impurity gradually decreases as the level of Sr addition gradually increases. The Ca/P ratio from the ICP-OES analysis and computational analysis of the XRD patterns using DIFFRAC.EVA are similar (shown in Table S1).

**Figure 4:**
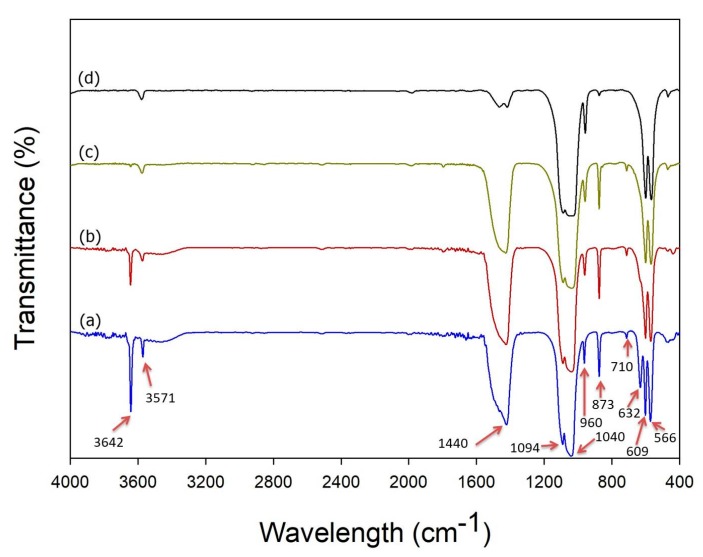
Fourier transform infrared spectroscopy (FTIR) spectra of (**a**) 0mSrHANFs, (**b**) 1mSrHANFs, (**c**) 2mSrHANFs, and (**d**) 3mSrHANFs.

Figure 4 shows a characteristic FTIR spectrum for the mSrHANFs. The peaks at approximately 566 and 609 cm^−1^ are due to the bending vibration of the P–O bond in PO_4_^3−^ [33]. The peaks noted at approximately 960 and 1000~1100 cm^−1^ are associated with the stretching modes of the PO_4_^3−^ bonds in HAp. [34] Moreover, the characteristic peaks observed at approximately 1440~1470 and 710 cm^−1^ are attributable to the CO_3_^−2^ group [35]. The intensity of the characteristic peak at 875 cm^−1^, assigned to the acidic phosphate group (HPO_4_^2−^) in HAp [36], gradually decreased as the doping amount of Sr increased. The characteristic peaks observed at 632 and 3571 cm^−1^ correspond to the stretching and bending vibration of the hydroxyl groups of HAp [37]. Frasnelli et al. [38] pointed out that strontium ions cause drastic changes in the local chemical environment of the apatitic functional groups and affect the hydroxyl vibrational mode, resulting in a reduction in the corresponding FT-IR absorption signal. Additionally, the sharp peak at 3642 cm^−1^, which confirms the formation of the CaO phase [39], gradually decreased as the doping amount of Sr increased. 

**Figure 5:**
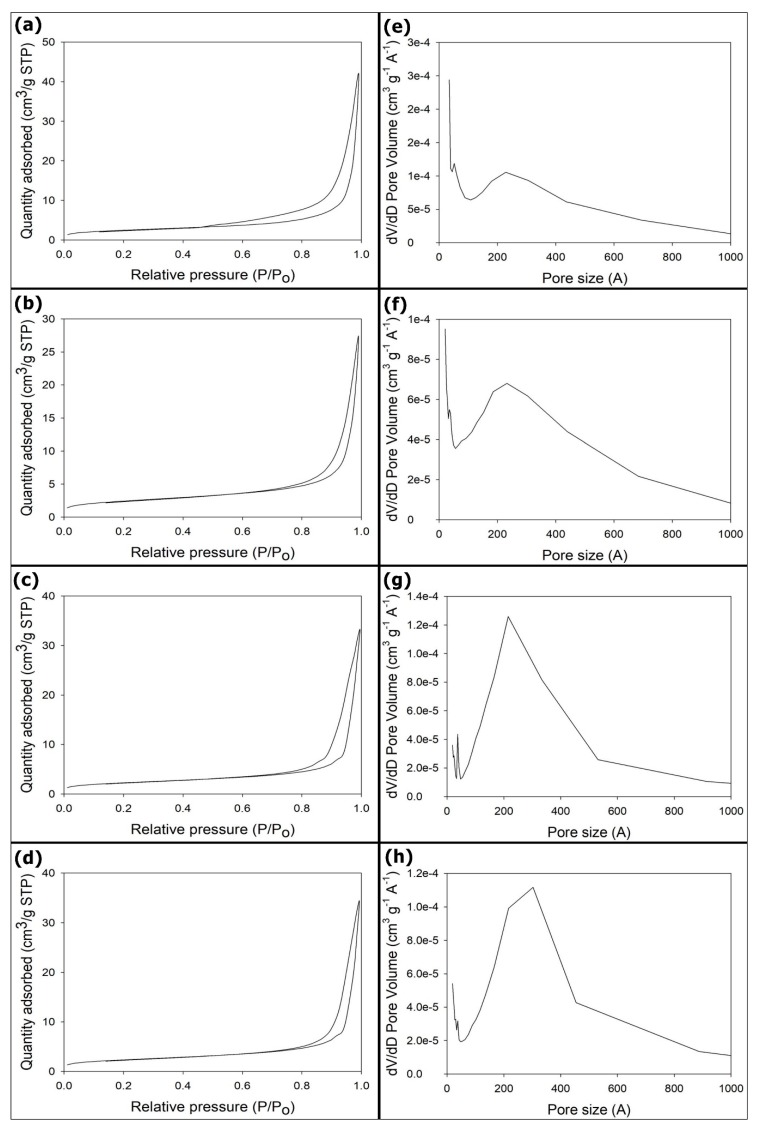
(**a**–**d**) N_2_ adsorption–desorption isotherm and (**e–f**) pore size distribution curve. (**a**,**e**) 0mSrHANFs; (**b**,**f**) 1mSrHANFs; (**c**,**g**) 2mSrHANFs; (**d**,**h**) 3mSrHANFs.

Fifteen milligrams of the samples were employed in the nitrogen adsorption–desorption experiments. The sample was tested once per batch. N_2_ adsorption–desorption isotherms for the mSrHANFs are presented in Figure 5. The shape of isotherms indicated that the pore structures of the all the mSrHANFs are approximately type IV isotherms. This isotherm type is characteristic of mesoporous materials according to the International Union of Pure and Applied Chemistry (IUPAC) classification. The BET specific surface areas and total pore volumes of all the mSrHANFs were approximately 8 m^2^/g and 0.05 cm^3^/g, respectively (shown in Table 5). The pore diameters exhibit a wider size distribution and a small population with a larger pore size, and the mean pore diameters for all the mSrHANFs were approximately 20~25 nm. Zhang et al. fabricated mesoporous strontium hydroxyapatite nanorods using CTAB as templates and found that the size of the mesopores was 3~5 nm. [19] We speculate that the larger pore size of the mSrHANFs may be caused by the pyrolysis of the polymers (PVP and P123) added to the precursor to increase the viscosity of the solution in the electrospinning process.

**Figure 6:**
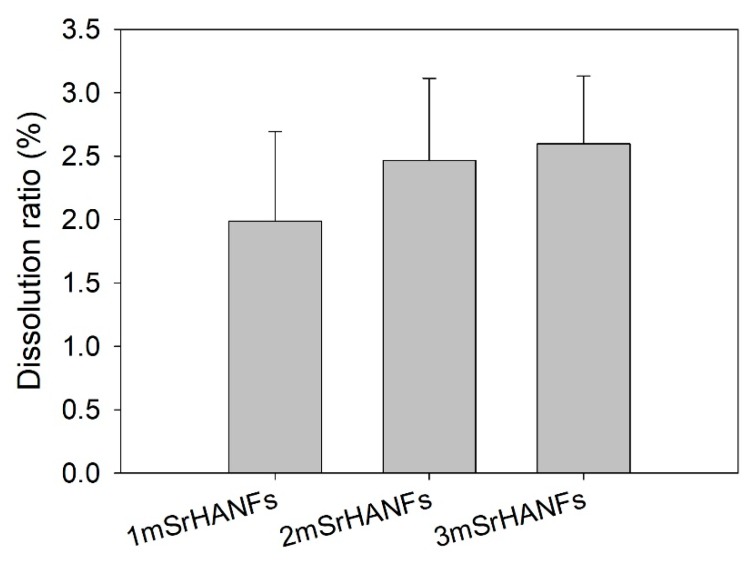
Degradation rate of the mesoporous strontium-substituted hydroxyapatite (mSrHANFs) after soaking in minimal essential medium (MEM) solution for 1 day. The data are presented as the averages ± standard derivations (n = 3).

Figure 6 shows the quantitative dissolution of the mSrHANFs after soaking in MEM solution for 1 day. The degradation ratios of the 1mSrHANFs, 2mSrHANFs, and 3mSrHANFs were 1.98%, 2.46%, and 2.60%, respectively. The results showed that the dissolution ratio increased as the doping amount of Sr increased.

**Figure 7:**
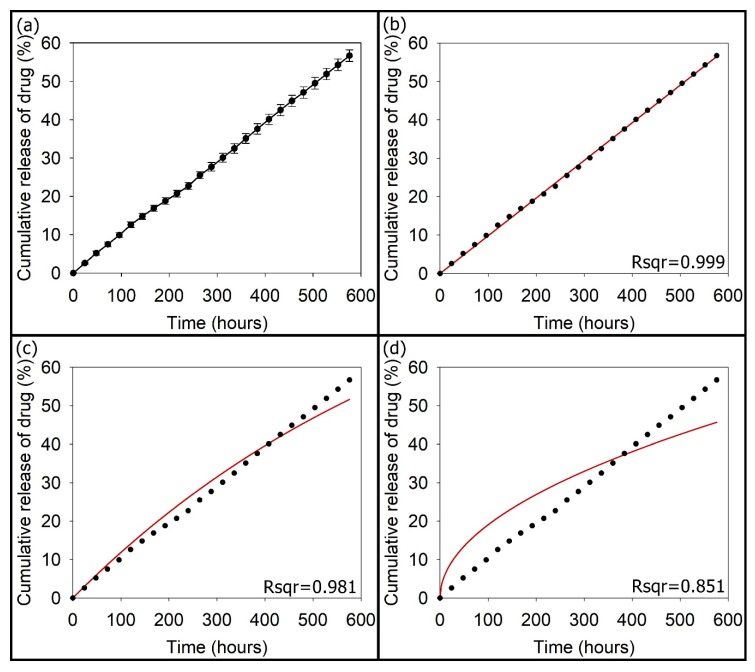
(**a**) In vitro cumulative tetracycline (TC) release from TC-loaded 3mSrHANFs. The curve was fitted with the equations of the (**b**) zero-order, (**c**) first-order, and (**d**) Higuchi models.

The amount of TC loaded within the 3mSrHANFs was 19.44 ± 0.15 mg/20 mg (TC/3mSrHANFs). The loading efficiency of TC was 97.21 ± 0.75% (*w*/*w*). Figure 7 shows the cumulative drug release profile as a function of time for TC release from the TC-loaded 3mSrHANFs. The release of TC from the TC-loaded 3mSrHANFs was not burst release at the initial period and remained steady and slow over 24 days. The release rate of TC from the TC-loaded 3mSrHANFs was approximately 2.36% per day. This rate is similar to the degradation rate of the 3mSrHANFs. The mechanism of drug release was analyzed using different kinetic models, i.e., the zero-order [Q_t_ = k_0_t], first-order [ln (Q_0_/Q_t_) = k_1_t], and Higuchi [Q_t_ = k_h_t^1/2^] models, to fit the drug release data. In these equations, k_0_, k_1_, and k_h_ are kinetic constant, Q_t_ is the cumulative amount of TC released at time t, and Q_0_ is the initial amount of TC present in the 3mSrHANFs. The best fit with the highest correlation coefficient was the zero-order equation (shown as Figure 7). 

The drug release data were further analyzed by the Ritger–Peppas equation (M_t_/M_∞_ = k_r_*·*t^n^), where M_t_ is the cumulative amount of drug released at time t, M_∞_ is the cumulative amount of drug released at time ∞, and k_r_ and n are the release rate constant and diffusion exponent, which were 0.0982 and 0.99, respectively. For the Ritger–Peppas model, 0.5 < n < 1.0 indicates anomalous diffusion, i.e., drug release that is both erosion and diffusion controlled. In our past research, the amount of TC loaded within the p-HApFs was 17.66 ± 0.18 mg/20 mg (TC/p-HApFs). The loading efficiency of TC was 88.34 ± 0.89% (*w*/*w*) [23]. The release rate constant was 0.846 for the release of TC from the TC-loaded p-HApFs. Comparison with the above results shows the 3mSrHANFs possess high loading efficiency and slow release of TC, which could be due to the strong interaction and complex formation between the TC molecules and strontium [40,41].

**Figure 8:**
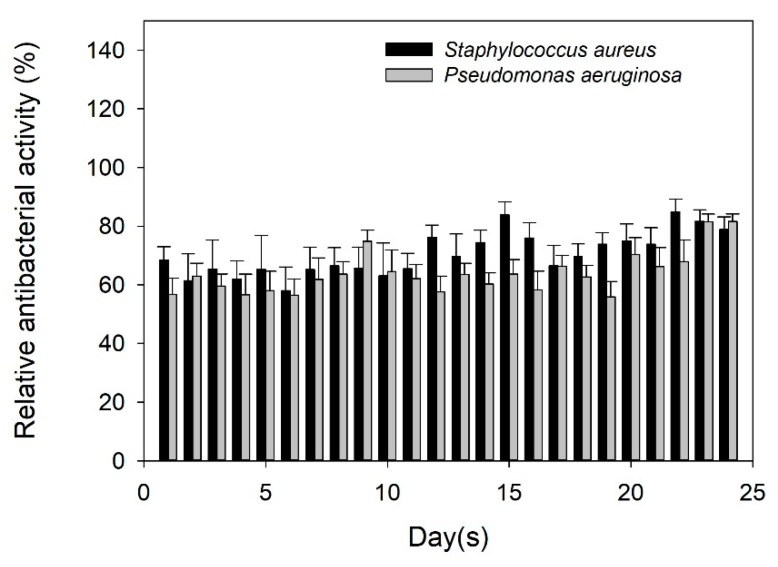
Susceptibility profiles of Staphylococcus aureus and Pseudomonas aeruginosa to released TC.

To clarify the antibacterial activity of TC released from the TC-loaded 3mSrHANFs, a release solution was used to cultivate bacterial strains. In addition, TC solution (0.15 mg/mL, free drug) was used as a negative control. The concentration of free TC is the same as the TC concentration from TC-loaded mSrHANFs. The results demonstrate that the relative antibacterial activity of free TC against the Gram-positive bacteria *Staphylococcus aureus* and the Gram-negative bacteria *Pseudomonas aeruginosa* are approximately 82% and 94%, respectively. The antibacterial activity of TC eluted from TC-loaded 3mSrHANFs was lower than that of free TC. TC inhibits bacterial growth by interfering with some metal-requiring step in the energy metabolism. Doluisio et al. noted that either a propanolamine-type complex (between the dimethylamino group and the C-12a hydroxyl group) or an ethanolamine-type complex (between the dimethylamino group and the C3 hydroxyl group) can form between the tetracyclines and divalent cations [42]. Lunestad et al. [43] found that the antibacterial activity of oxytetracycline is reduced in seawater, and this inhibition is due to the formation of a complex of the antibiotic chelated to divalent cations. We speculate that the Sr-TC complex was released from TC-loaded mSrHANFs. However, the solution obtained from TC-loaded 3mSrHANFs has the ability to effectively retard bacterial growth even on day 24 (Figure 8). Hence, TC-loaded 3mSrHANFs possess long-acting antibacterial activity.

## 4. Conclusions

Strontium-substituted hydroxyapatite-CaO-CaCO_3_ nanofibers containing mesoporous structures (mSrHANFs) were successfully fabricated from sol–gel precursors using the electrospinning method, and up to 30 mol% strontium could be successfully doped into the hydroxyapatite (HAp) structures. The contents of CaO and CaCO_3_ in the mSrHANFs decreased as the doping amount of strontium increase. Furthermore, the mSrHANFs possessed excellent drug loading efficiency and ability to release tetracycline (TC) in a sustained manner, leading to the maintenance of antibacterial activity over 3 weeks. Hence, mSrHANFs are suitable as bone grafts and as a drug carrier for repairing bone defects.

## Figures and Tables

**Table 1:** Sample notation and molar ratio for the fabrication of strontium-substituted hydroxyapatite nanofibers with a mesoporous structure (mSrHANFs).

Sample Notation	Molar Ratio (Sr/Ca)	Molar Ratio (Sr + Ca)/P
0mSrHANFs	0	1.67
1mSrHANFs	1/9	1.67
2mSrHANFs	2/8	1.67
3mSrHANFs	3/7	1.67

**Table 2:** Elemental composition of different samples measured by inductively coupled plasma–optical emission spectrometry (ICP-OES).

Sample Name	Nominal Sr/(Sr + Ca) (%)	Measured Sr/(Sr + Ca) (%)	Nominal (Sr + Ca)/P	Measured (Sr + Ca)/P
0mSrHANFs	0	0	1.67	3.17
1mSrHANFs	10	9.25	1.67	2.08
2mSrHANFs	20	18.94	1.67	1.73
3mSrHANFs	30	29.4	1.67	1.68

**Table 3:** Lattice constants, interplanar spacing for (211) and (002) diffraction of the mSrHANFs. (a and c: lattice constants; d:interplanar spacing).

Sample Name	Lattice Constants		d_(002)_ (Å)	d_(211)_ (Å)
	a (Å)	c (Å)		
0mSrHANFs	9.72	7.02	3.51	2.90
1mSrHANFs	9.80	7.06	3.53	2.92
2mSrHANFs	9.83	7.08	3.54	2.93
3mSrHANFs	9.90	7.12	3.56	2.95

**Table 4:** Phase composition of samples determined by DIFFRAC.EVA analysis of X-ray diffraction (XRD) patterns.

Sample Name	Ca_10-x_Sr_x_(PO_4_)_6_(OH)_2_ (%)	CaO (%)	CaCO_3_ (%)
0mSrHANFs	66.3	21.1	12.6
1mSrHANFs	77.5	21.5	1.0
2mSrHANFs	83.4	15.8	0.8
3mSrHANFs	96.1	1.4	2.5

**Table 5:** BET (Brunauer-Emerett-Teller) surface area, BJH (Barrett-Joyner-Halenda) total pore volume and pore size of mesoporous strontium-substituted hydroxyapatite (mSrHANFs).

Sample Name	Surface Area (m^2^/g)	BJH Total Pore Volume (cm^3^/g)	Average Pore Diameter (nm)
0mSrHANFs	8.62	0.065	21.8
1mSrHANFs	8.41	0.043	21.2
2mSrHANFs	7.87	0.052	25.1
3mSrHANFs	8.11	0.053	25.9

## Supplementary Materials

Supplementary File 1
