# Supplementary material for: Fabrication and Characterization of Strontium-Substituted Hydroxyapatite-CaO-CaCO3 Nanofibers with a Mesoporous Structure as Drug Delivery Carriers
Source: Pharmaceutics. 2018 Oct 8;10(4):179. doi: 10.3390/pharmaceutics10040179 (PMC6321556; doi:10.3390/pharmaceutics10040179)
Supplement: Supplementary File 1 [file pharmaceutics-10-00179-s001.pdf]

Table S1: The Ca/P ratio from the ICP-OES, SEM-EDS and computational mathematical analysis

| Sample name | ICP-OES Ca/P ratio | SEM-EDX Ca/P ratio | DIFFRAC.EVA Ca/P ratio |
|---|---|---|---|
| 0mSrHANFs | 3.17 | 4.21 | 2.96 |
| 1mSrHANFs | 2.08 | 2.83 | 2.51 |
| 2mSrHANFs | 1.73 | 2.16 | 2.24 |
| 3mSrHANFs | 1.68 | 2.25 | 1.75 |
